# Supplementary material for: Analysis of Antimicrobials in Muscle and Drinking Water in Terms of Reducing the Need of Antimicrobial Use by Increasing the Health and Welfare of Pig and Broiler
Source: Antibiotics (Basel). 2023 Feb 4;12(2):326. doi: 10.3390/antibiotics12020326 (PMC9952824; doi:10.3390/antibiotics12020326)
Supplement: Supplementary file 1 [file antibiotics-12-00326-s001.zip › antibiotics-2181554-supplementary.pdf]

Table S1. Biosecurity measures on broiler farms

Internal biosecurity measures	External biosecurity measures
- Create clearly marked 'traffic routes' between the red, orange and green zones.<br>- Placement of hand sanitizers at the entrance of each shed on the farm<br>- Placement of disinfection points (footpads) outside each shed<br>- Better disinfection of each shed between flocks after wet cleaning.<br>- Disinfection of all feed silos when completely empty between flocks.<br>- Systematic use of an water acidifier at specific points in the growth cycle - from starter feed to grower - from grower to finisher 1 and from finisher 1 to finisher 2<br>- Flushing of the water pipes inside the shed at preagreed frequency during each flock's growing period. Recording of such action in the shed diary.<br>- Install farm specific system for the oncall supply of hot water for the appropriate farm specific use during cleaning times between flocks<br>- Dry disinfection of the litter during the growing period should it be required. Implementing a pest managing program on a monthly basis.<br>- Chlorination of farm water at the point of entry if continued with the well or use from public human supply.<br>- Improve the current system for cleaning and disinfecting after each use of the collecting crates.	- Frequent and recorded use of disinfection by spraying of the immediate area outside each shed's entrance<br>- Creation of a plant free zone (3m) or a cemented & plant free zone (3m) surrounding the specific shed.<br>- Establish automatic disinfection pits and spraying facilities at the entrance of each farm.<br>- Training of catching teams against a protocol of all aspects of biosecurity relevant to their jobs & change of way of catching, enhancing this way the welfare of the birds<br>- Placement of appropriate closed and secure points for the collection of all daily mortalities and culls at the exit point of the whole farm.<br>- Build/create a separate and closed entry room for each shed.<br>- Retraining of all farm workers on the key aspects of biosecurity - COVID pandemic was an opportunity of improvement<br>- Cleaning and disinfection of all water storage tanks on the farm as well as the water pipes outside the sheds at least once between flocks.

Table S2. Biosecurity measures on pig farms

Biosecurity measures
- Known health status of purchased breeding pigs, coming from farms free of specific diseases (Specific Pathogen Free farms)<br>- Provision of adequate filter zones (hygiene lock) at the entrance of pig houses where farm workers and visitors can change clothes and footwear and disinfect hands and shoes, with strict separation between dirty and clean area.<br>- Purchase of a carcass cooled storage dedicated to the collection of dead pigs, physically separated from animal facilities (placed in the dirty area of the farm)<br>- Provision of specific gloves, clothing and footwear to personnel in charge of removing and transferring dead animals into the carcass storage cell<br>- Adoption of a specific protocol for the cleaning and disinfection of the equipment after their use and of the room where it is stored<br>- Continuous training of farm workers on biosecurity standard procedures<br>- Use of compartment specific veterinary equipment (i.e. needles)
